# Transcriptomic response of *Pseudomonas nicosulfuronedens* LAM1902 to the sulfonylurea herbicide nicosulfuron

**DOI:** 10.1038/s41598-022-17982-7

**Published:** 2022-08-11

**Authors:** Miaomiao Li, Qingqing Li, Jun Yao, Geoffrey Sunahara, Robert Duran, Qinghua Zhang, Zhiyong Ruan

**Affiliations:** 1 School of Water Resource and Environment, Research Center of Environmental Science and Engineering, China University of Geosciences (Beijing), 29 Xueyuan Road, Haidian District, Beijing, 100083 China; 2 Institute of Agricultural Resources and Regional Planning, Chinese Academy of Agricultural Sciences, Beijing, 100081 China; 3 Department of Natural Resource Sciences, McGill University, 21111 Lakeshore Drive, Ste-Anne-de-Bellevue, Quebec, H9X 3V9 Canada; 4 College of Bioscience and Engineering, Jiangxi Agricultural University, Nanchang, 330045 People’s Republic of China; 5 Universite de Pau Et Des Pays de L’Adour, UPPA/E2S, IPREM CNRS 5254, Pau, France

**Keywords:** Microbiology, Molecular biology

## Abstract

The overuse of the herbicide nicosulfuron has become a global environmental concern. As a potential bioremediation technology, the microbial degradation of nicosulfuron shows much promise; however, the mechanism by which microorganisms respond to nicosulfuron exposure requires further study. An isolated soil-borne bacteria *Pseudomonas nicosulfuronedens* LAM1902 displaying nicosulfuron, chlorimuron-ethyl, and cinosulfuron degradabilities in the presence of glucose, was used to determine the transcriptional responses to nicosulfuron exposure. RNA-Seq results indicated that 1102 differentially expressed genes (DEGs) were up-regulated and 702 down-regulated under nicosulfuron stress. DEGs were significantly enriched in “ABC transporters”, “sulfur metabolism”, and “ribosome” pathways (*p* ≤ 0.05). Several pathways (glycolysis and pentose phosphate pathways, a two-component regulation system, as well as in bacterial chemotaxis metabolisms) were affected by nicosulfuron exposure. Surprisingly, nicosulfuron exposure showed positive effects on the production of oxalic acid that is synthesized by genes encoding glycolate oxidase through the glyoxylate cycle pathway. The results suggest that *P. nicosulfuronedens* LAM1902 adopt acid metabolites production strategies in response to nicosulfuron, with concomitant nicosulfuron degradation. Data indicates that glucose metabolism is required during the degradation and adaptation of strain LAM1902 to nicosulfuron stress. The present studies provide a glimpse at the molecular response of microorganisms to sulfonylurea pesticide toxicity and a potential framework for future mechanistic studies.

## Introduction

China is a large agricultural country that annually produces and consumes hundreds of different pesticides^1^. Nicosulfuron, a typical sulfonylurea herbicide, has been widely used to eradicate different types of broadleaf grasses and weeds in agriculture^2–4^. Nicosulfuron inhibits plant acetolactate synthase, a key feedback enzyme associated with the synthesis of branched-chain amino acids such as leucine, valine, and isoleucine^5–7^. Inhibited synthesis of these amino acids can lead to a rapid decrease in cell division and plant growth. Due to the extensive use of nicosulfuron in agriculture and the persistence of residuals in farmland soil, nicosulfuron can be acutely toxic to susceptible crops and a decrease in biodiversity^8–12^. Preliminary studies showed that nicosulfuron decreased wheat seed germination and emergence as low as 2 mg/kg (Li, pers. communication). Residual herbicides or their intermediates can pose severe ecotoxicological risks, such as altering the soil microbial community, and lethality to some aquatic organisms (e.g., fish and crayfish)^13–19^. Furthermore, the use of multiple herbicides is a common practice in the real environment, increasing the risk of herbicides to the ecological environment^18^. It is therefore important to understand and evaluate the potential environmental risks of nicosulfuron exposure and gain greater insight into its degradation mechanisms.

The degradation of nicosulfuron by chemical hydrolysis, photolysis, and microbial activity has been reported^20–23^. Microbial degradation is considered an environmentally friendly technology to mitigate hazardous residual concentrations of sulfonylurea herbicides in the environment^24–26^. Many bacteria and fungi can harbor nicosulfuron degradation ability, including *Alcaligenes faecalis* ZWS11^27^, *Bacillus subtilis* YB1^28^, *Klebsiella* sp. Y1^29^, *Oceanisphaera psychrotolerans* LAM-WHM-ZC^30^, *Aspergillus niger* YF1^31^, *Talaromyces flavus* LZM1^32^, *Penicillium oxalicum* YC-WM1^33^, and *Plectosphaerella cucumerina* AR1^34^. *Pseudomonas nicosulfuronedens* LAM1902 (JCM33860, KCTC72830) (hereafter referred to as strain LAM1902) is a recently identified nicosulfuron-degrading aerobic gram-negative bacterium having motile short rods with a cell width of 0.5–0.7 mm and length of 0.8–1.1 mm^35^. Most of these strains transform nicosulfuron into 2-(aminosulfonyl)-N,N-dimethyl-3-pyridinecarboxamide (ASDM) and 2-amino-4,6-dimethoxypyrimidine (ADMP)^34^. Li et al.^35^ proposed a self-protection mechanism in strain LAM1902 under nicosulfuron stress using a non-targeted metabolomics approach; however, the molecular mechanism(s) underlying microbial nicosulfuron degradation requires further elucidation.

The development of advanced sequencing technologies allows the investigation of molecular mechanisms involved in the detoxification and adaptation of microbial strains to environmental pollutant exposure^36–38^. For example, transcriptomic analysis using differentially expressed genes (DEGs) has been used to examine the gene responses to various environmental stimuli, particularly for the elucidation of genes involved in degradation^39–41^. Transcriptome sequencing can provide strong support for identifying genes involved in pollutant degradation by microorganisms^42^. This technique has been used to investigate the degradation mechanism(s) of sulfonylurea herbicides. For example, RNA-Seq analysis showed that chlorimuron-ethyl (a pro-herbicide for chlorimuron) degradation by *Rhodococcus erythropolis* D310-1 was associated with increased cytochrome P-450, carboxylesterase, and monooxygenase^41^. To the best of our knowledge, there are no published reports describing the use of transcriptomic analyses for identifying the molecular mechanism(s) of nicosulfuron-degrading bacterial strains.

In the present study, we hypothesize that strain LAM1902 survives nicosulfuron stress by developing adaptive mechanism(s) favoring nicosulfuron degradation. The specific aims of this study were to: (1) investigate the degradation conditions of nicosulfuron by strain LAM1902; (2) use the transcriptome sequencing approach to identify the key metabolic pathways involved in the transcription process of strain LAM1902 following nicosulfuron exposure, and (3) elucidate the adaptive mechanisms of strain LAM1902 to nicosulfuron exposure.

## Results and discussion

### Optimization of the nicosulfuron degradation conditions for LAM1902

**Figure 1:**
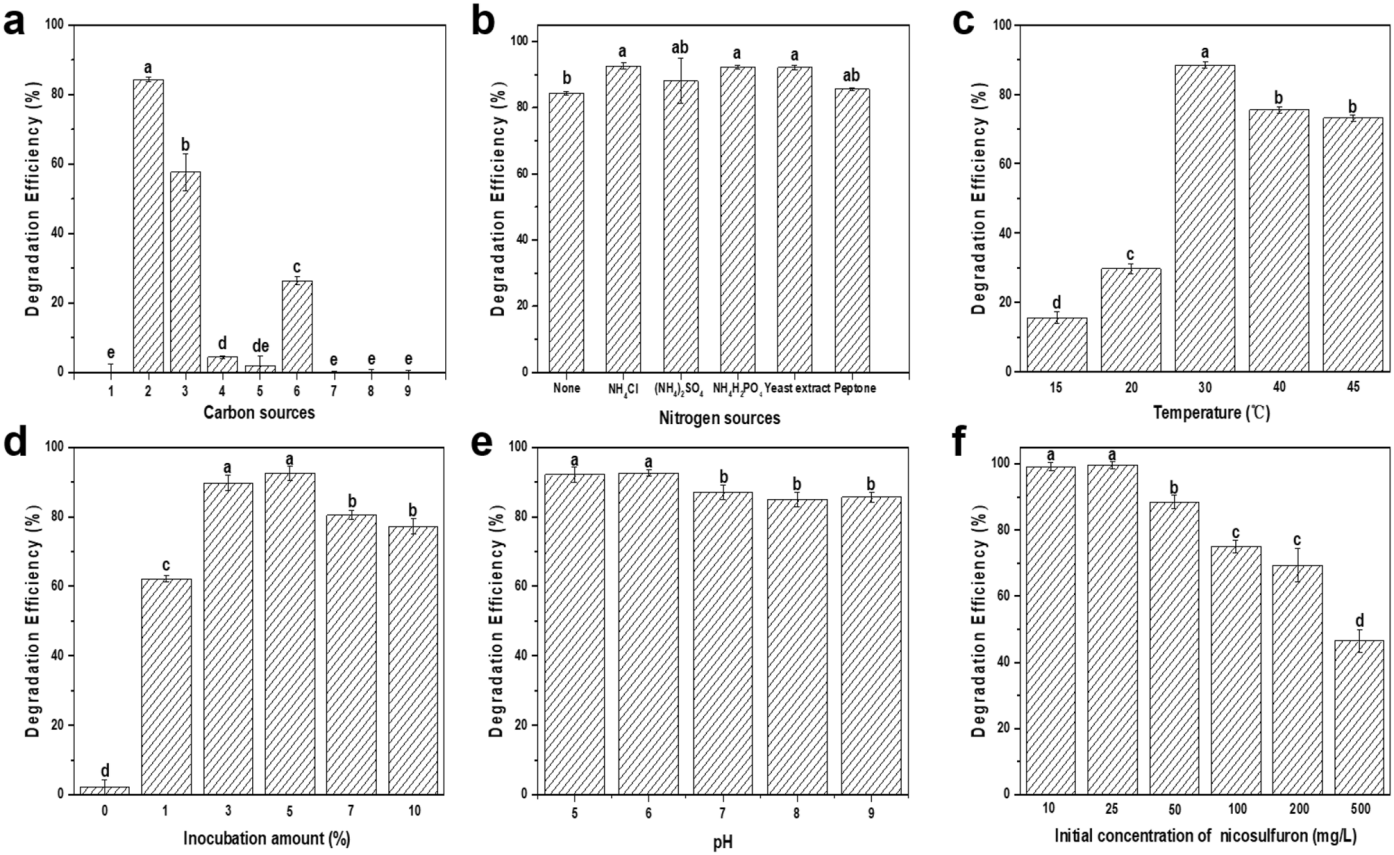
Degradation of nicosulfuron by *Pseudomonas nicosulfuronedens* LAM1902 under different conditions after 6 days of incubation. (**a**) Carbon sources (1–9 represent none, glucose, glycerol, sodium acetate, sodium succinate, yeast extract, peptone, sucrose, starch, respectively); (**b**) nitrogen sources; (**c**) temperature (°C), (**d**) incubation amount (%), (**e**) different initial pHs (5, 6, 7, 8, and 9), and (**f**) initial concentration of nicosulfuron (mg/L). Data expressed as average ± SD (n = 3). Common letters denote no significant difference (*p* > 0.05) between treatment groups (Waller-Duncan posthoc test).

The effects of various factors (including the different C- and N- sources, pH (5–9), temperature (30–45 °C), and the initial incubation volume) on nicosulfuron degradation by LAM1902 were determined after 6 days of incubation (Fig. 1). As shown in Fig. 1a, the nicosulfuron degradation efficiency of strain LAM1902 was 84% in the presence of glucose, higher than other carbon sources (*p* ≤ 0.05). It is assumed that the preference for C-source was related to the metabolic system of LAM1902, so glucose was selected as the C-source in the following studies. There were no significant effects on nicosulfuron degradation efficiency among different nitrogen sources (NH_4_Cl, (NH_4_)_2_SO_4_, NH_4_H_2_PO_4_, yeast extract, and peptone; *p* > 0.05). It is worth noting that LAM1902 can use nicosulfuron as the sole nitrogen source. Therefore, nicosulfuron may provide sufficient N for LAM1902 growth in the absence of a supplementary N- source (Fig. 1b). Optimal conditions for nicosulfuron degradation by LAM1902 were at 30 °C, pH 5–6, and inoculum biomass at 5.0% (v/v) (Fig. 1c–e). The degradation efficiency of nicosulfuron was not significantly different (*p* > 0.05) between pH 5 and pH 6. It was noteworthy that the pH decreased from pH 7.0 to pH 3.5 during the degradation process by LAM1902, likely due to the production of organic acids. Concentration–response studies showed that the nicosulfuron degradation efficiency of strain LAM1902 was 99% after 6 days of incubation using 25 mg/L nicosulfuron, whereas incubation at 500 mg/L (the highest concentration tested) was toxic to the microbes as evidenced by a decrease (47%) in the nicosulfuron degradation efficiency (Fig. 1f). The extrapolated IC50 (based on the three highest concentrations) for degradation efficiency was 457.0 mg/L (Fig. S1).

**Figure 2:**
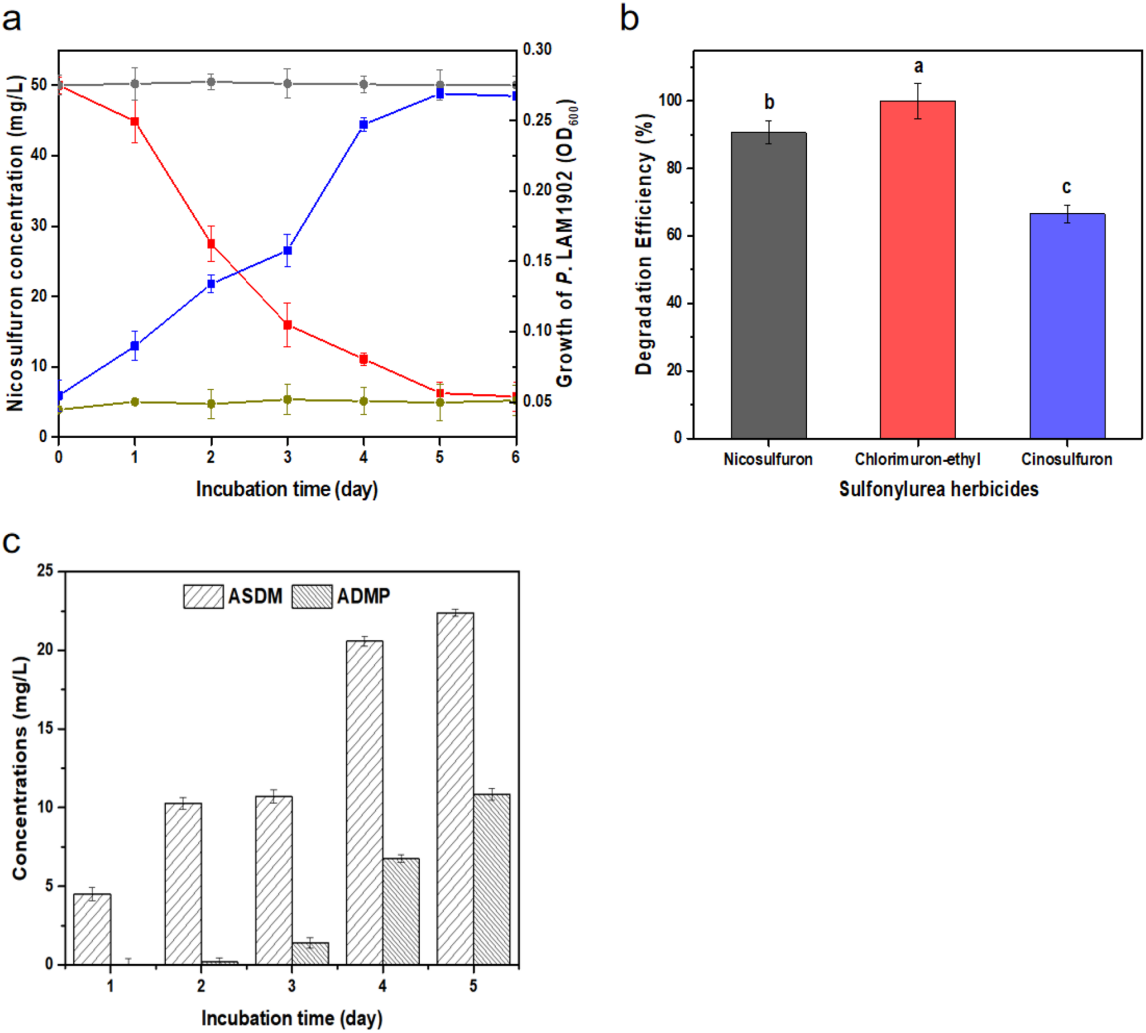
(**a**) Time course of nicosulfuron (NS) degradation  and growth curve  for *Pseudomonas nicosulfuronedens* LAM1902 live and dead, respectively. (**b**) Comparison of the degradation percentage of nicosulfuron (black bar), chlorimuron-ethyl (red bar) and cinosulfuron (blue bar) (50 mg/L) by LAM1902 after 6 days of incubation. Common letters above bars denote no significant difference (*p* > 0.05) between treatment groups. Data expressed as average ± SD (n = 3). (**c**) Time course of metabolite formation during nicosulfuron degradation: ASDM (2-(aminosulfonyl)-N,N-dimethyl-3-pyridinecarboxamide) and ADMP (2-amino-4,6-dimethoxypyrimidine).

Data indicates that the 6 days nicosulfuron degradation efficiency of strain LAM1902 was 95%, with a cell density OD_600_ of 0.27 (Fig. 2a). The metabolites (ASDM and ADMP) were also detected during nicosulfuron degradation (Fig. 2c). Strain LAM1902 showed similar efficiencies to degrade other sulfonylurea herbicides, such as chlorimuron-ethyl and cinosulfuron (Fig. 2b). Chlorimuron-ethyl is a selective post-emergence herbicide for controlling actively growing weeds in peanut, soybeans, and non-crop areas. Cinosulfuron is a broad spectrum triazinylsulfonylurea herbicide used for post-emergence control of many weeds, including European water plantain, annual sedge, aquatic ferns, and pod weeds. These herbicides are highly water-soluble and can leach from soil to groundwater and surface water. According to the molecular structure of the three sulfonylurea herbicides (Fig. S2), these share a similar structure, a urea bridge, which suggests that LAM1902 hydrolyzes the urea bridge in nicosulfuron as well as other sulfonylurea herbicides (chlorimuron-ethyl and cinosulfuron). The broad spectrum of sulfonylurea herbicide degradation by LAM1902 can be used to develop microbial strategies or mechanisms mitigating the toxic effects of sulfonylurea herbicides on the environment. As a first approach to investigate these mechanisms, studies were undertaken to determine the transcriptomic response of strain LAM1902 to nicosulfuron exposure.

### RNA-Seq analysis of the strain LAM1902 transcriptome during nicosulfuron degradation

The Illumina Hiseq Xten sequencing provided a total of 10,277,078 and 10,166,956 clean reads for the YG experimental (using nicosulfuron) and NG control (without nicosulfuron) groups, respectively. More than 98% of transcripts had a size above 20 bp in both groups. The YG and NG groups showed ≥ 94% and ≥ 96% of specific sequences, respectively (Table S3). Principal component analysis (PCA) confirmed the differences between the treated (under nicosulfuron) and control (without nicosulfuron) groups (Fig. S3). The analyses of these transcripts were used to identify genes involved in nicosulfuron biodegradation and better understand the mechanisms underlying the microbial response(s) to nicosulfuron.

### Differential gene expression of strain LAM1902 in response to the presence of nicosulfuron

**Figure 3:**
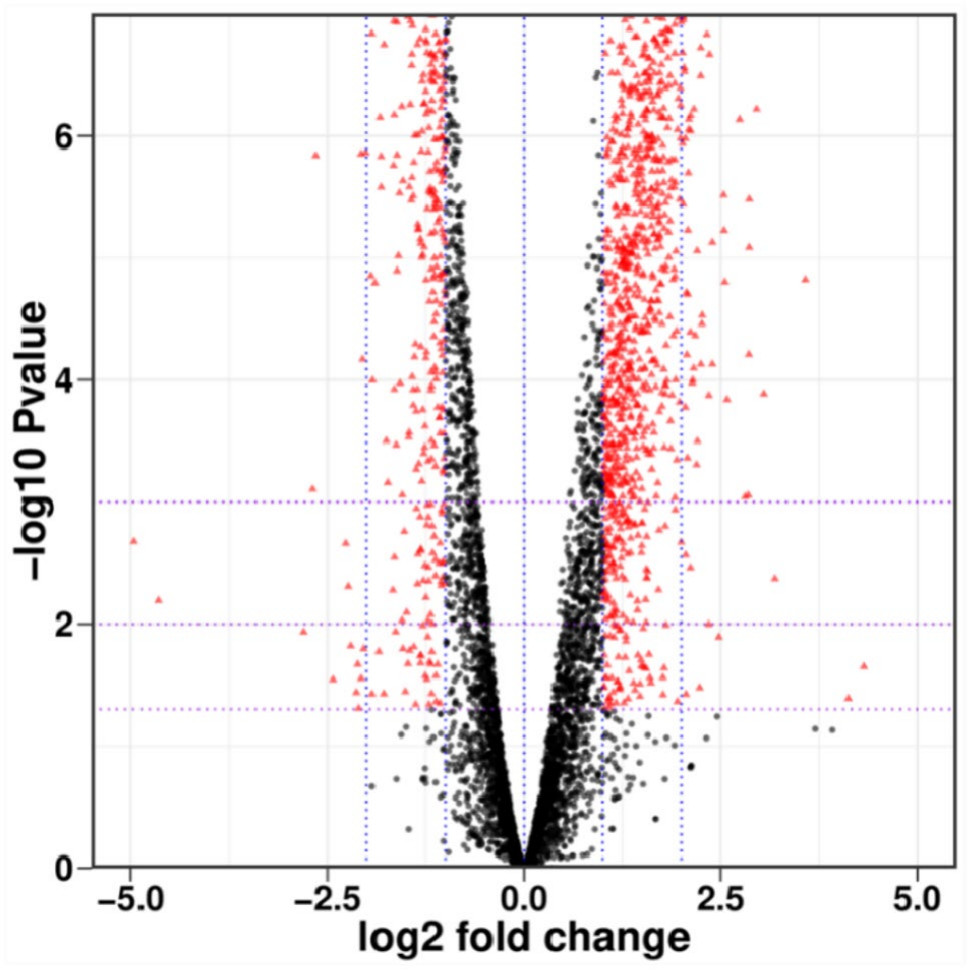
Volcano map showing differentially expressed genes (DEGs) comparing the control group (NG, LAM1902 without nicosulfuron in GSM) to the treated group (YG, LAM1902 in the presence of 50 mg/L nicosulfuron in GSM) (*p* ≤ 0.05 and | log_2_(fold change) |≥ 1). The DEGs that are up-regulated or down-regulated are colored in red, and the undifferentiated genes expressed in two groups were colored in black.

A total of 6021 expressed genes were identified. Among these, 1804 genes were considered as DEGs (*p* ≤ 0.05 and |log_2_(fold change)|≥ 1) in the treated (under nicosulfuron) and control (without nicosulfuron) groups, including 1102 up-regulated DEGs (61%) and 702 down-regulated DEGs (39%) (Fig. 3). The up-regulated expression genes might be related to the response strategy of nicosulfuron, while down-regulated genes (39%) suggested that nicosulfuron still had toxic effects on strain LAM1902^43,44^. The distribution of the top 30 most expressed genes in both groups is shown in Fig. S4.

### GO and KEGG enrichment analyses of DEGs

The differentially expressed genes (DEGs) were annotated and classified according to the GO and KEGG databases to determine the metabolic processes of LAM1902 induced in response to nicosulfuron exposure. The DEGs were distributed as “cell part”, “catalytic activity”, “cellular process”, “metabolic process”, and “binding” categories based on the GO database (Fig. S5). According to the KEGG database, a total of 757 DEGs were dispatched within 200 pathways, including “environmental information processing”, “cellular processes”, “genetic information processing”, “human diseases”, “metabolism”, and “organismal systems.” Among these, “metabolism” included the main (57%) metabolic pathways (in KEGG A class), whereas a total of 159 and 89 DEGs were related to the “carbohydrate metabolism” and “xenobiotics biodegradation and metabolism”, respectively.

**Figure 4:**
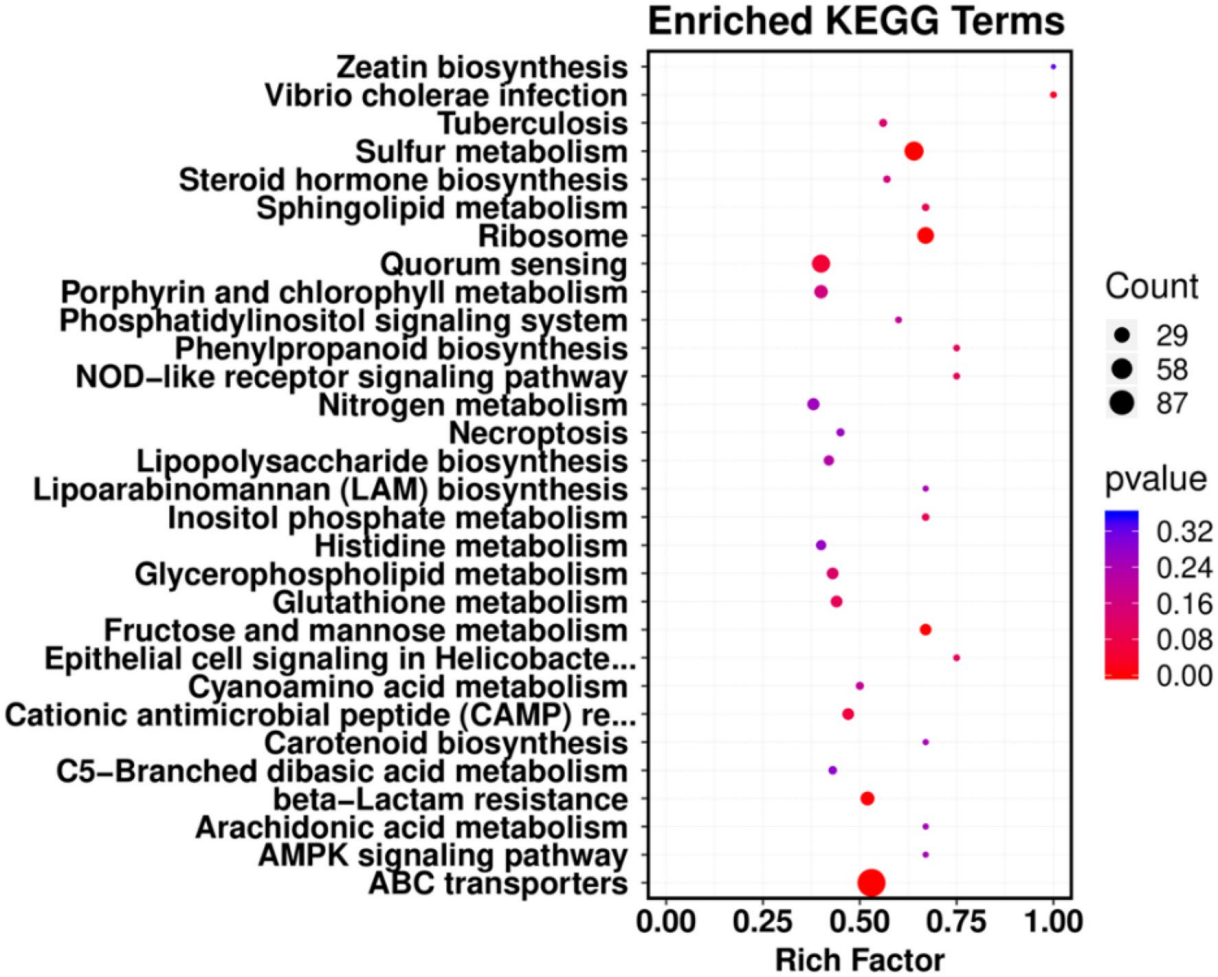
The differentially expressed genes enriched in KEGG pathways during nicosulfuron degradation by *Pseudomonas nicosulfuronedens* LAM1902^45^.

According to the KEGG enrichment analyses^45^, DEGs were significantly enriched in “ABC transporters”, “sulfur metabolism”, and “ribosome” pathways (*p* ≤ 0.05) (Fig. 4 and Table S4), belonged to “membrane transport”, “translation”, and “energy metabolism” categories, respectively. A total of 115, 49, and 36 DEGs respectively were associated with these pathways. All DEGs involved in the “ribosome” pathway suggested that nicosulfuron had detrimental effects on LAM1902 ribosomal pathways. In contrast, DEGs involved in “ABC transporters” (94 up-regulated and 21 down-regulated genes) and many genes involved in “sulfur metabolism” (44 genes up-regulated and five down-regulated genes) pathway were generally up-regulated after nicosulfuron exposure. The “ABC transporters” can transform and transport toxic substances through the cell membrane to support cellular defense systems^46–48^. This pathway also plays an important function in drug resistance, metabolism, and toxicity^49^. A highly efficient degradation enzyme of nicosulfuron, the manganese ABC transporter, was purified from strain *Bacillus subtilis* YB1^50,51^. In addition, the “manganese ABC transporters” can degrade nicosulfuron through the strong hydrophobic interactions and hydrogen bonds^52^. Furthermore, sulfur is an important nutrient for the growth of microorganisms and participates in electron transport and cell regulation. Our previous study showed that the degradation of nicosulfuron was due to the cleavage of the carbon–sulfur bond (C–S) in the urea bridge^35^. Therefore, “sulfur metabolism” was an important pathway in the degradation process of nicosulfuron. The above results revealed that the DEGs involved in “ABC transporters” and “sulfur metabolism” pathways were the more reactive pathways and were stimulated by nicosulfuron. These pathways likely play vital roles in the response of LAM1902 to nicosulfuron stress.

### Metabolic pathway analysis of the response of strain LAM1902 to nicosulfuron stress

Earlier studies have suggested that nicosulfuron degradation could be accomplished using co-supplementation with C-^42^, promoting microorganisms to survive in the nicosulfuron-containing culture/incubation medium. In the present study, the biodegradation of nicosulfuron was carried out in the presence of glucose, and the supplementation of this carbon source was quite effective. Many DEGs in strain LAM1902 were involved in glucose metabolism (based on KEGG database annotation) and included glycolysis, gluconeogenesis, pentose phosphate pathway, glyoxylate, and dicarboxylate metabolism, tricarboxylic acid (TCA) cycle, and other major metabolism pathways (Table 1). These pathways might be important in cellular adaptation to nicosulfuron stress^41^.

**Table 1:** KEGG classification of differentially expressed genes during nicosulfuron biodegradation in *Pseudomonas nicosulfuronedens* LAM1902.

KEGG annotation	A_Class	B_Class	Up-related genes	Down-related genes	ko_ID
ABC transporters	Environmental Information Processing	Membrane transport	94	21	ko02010
Sulfur metabolism	Metabolism	Energy metabolism	44	5	ko00920
Ribosome	Genetic Information Processing	Translation	0	36	ko03010
Carbon fixation in photosynthetic organisms	Metabolism	Energy metabolism	1	2	ko00710
Carbon fixation pathways in prokaryotes	Metabolism	Energy metabolism	3	7	ko00720
Central carbon metabolism in cancer	Human Diseases	Cancers: Overview	2	0	ko05230
One carbon pool by folate	Metabolism	Metabolism of cofactors and vitamins	1	1	ko00670
Nitrogen metabolism	Metabolism	Energy metabolism	13	3	ko00910
Citrate cycle (TCA cycle)	Metabolism	Carbohydrate metabolism	0	10	ko00020
Pyruvate metabolism	Metabolism	Carbohydrate metabolism	12	3	ko00620
Glycolysis/Gluconeogenesis	Metabolism	Carbohydrate metabolism	5	5	ko00010
Pentose phosphate pathway	Metabolism	Carbohydrate metabolism	5	3	ko00030
Fatty acid biosynthesis	Metabolism	Lipid metabolism	7	6	ko00061
Fatty acid degradation	Metabolism	Lipid metabolism	12	8	ko00071
Two-component system	Environmental Information Processing	Signal transduction	54	40	ko02020
Bacterial chemotaxis	Cellular Processes	Cell motility	8	1	ko02030

Twelve up-regulated DEGs were involved in pyruvate metabolism in the YG (in the presence of nicosulfuron) groups (ko00620), which can catalyze the ADP to ATP and enhance the glycolysis activity^53^. During the metabolism of glycolysis, one mole of glucose can be broken down into two moles of pyruvate, which could be metabolized to acetyl-CoA under aerobic conditions. The acetyl-CoA can enter the TCA cycle, in which organisms can obtain energy in response to the nicosulfuron stress^54^. Ten DEGs involved in the glycolysis or gluconeogenesis pathway were identified (Table 1). Under exposure to nicosulfuron, strain LAM1902 can alter its activities during glycolysis and the TCA cycle as an adaptation to environmental stress. Similar changes in glycolysis have been found in other microorganisms exposed to environmental stress^55^.

The pentose phosphate pathway involves the oxidative decomposition of glucose, which can be directly oxidatively dehydrogenated and decarboxylated^56^. It is a common carbohydrate catabolic pathway in microbes, animals, and plants^57^. In addition to the energy provided by the pentose phosphate pathway, some intermediate products are generated from this pathway that can provide substrates for the biosynthesis of many substances, such as 5-P-ribose, nucleotides, 4-P-erythrose, and aromatic amino acids. On the other hand, this pathway also produces a large amount of NADPH for the synthesis of fatty acids and sterols^42^. In the present study, five up-regulated DEGs of the pentose phosphate pathway (ko00030) were identified, which may be responsible for providing NADPH for amino acid and fatty acid metabolism in strain LAM1902. This is interesting to note that the DEGs related to fatty acid degradation were higher than the fatty acid synthesis under nicosulfuron stress. These results showed that the metabolism of glucose was affected under the exposure of nicosulfuron, which might be related to the adaptation strategy of strain LAM1902 to environmental stress. Furthermore, a prominent finding of the present study was the enhanced expression of a two-component regulation metabolism involving a total of 94 DEGs, based on the KEGG database (Table 1). The two-component signal transduction system enables microorganisms to sense, respond, and adapt to the changes in many different environmental conditions, thereby allowing strains to make a corresponding stress response to maintain their health under harsh conditions. Ganesh et al.^58^ reported that the abundance of genes associated with the two-component regulation system was important for microorganisms to survive in harsh environments. Overall, the enhancement of the two-component regulation process in strain LAM1902 likely underlies (at least in part) the adaptation process to nicosulfuron exposure.

**Figure 5:**
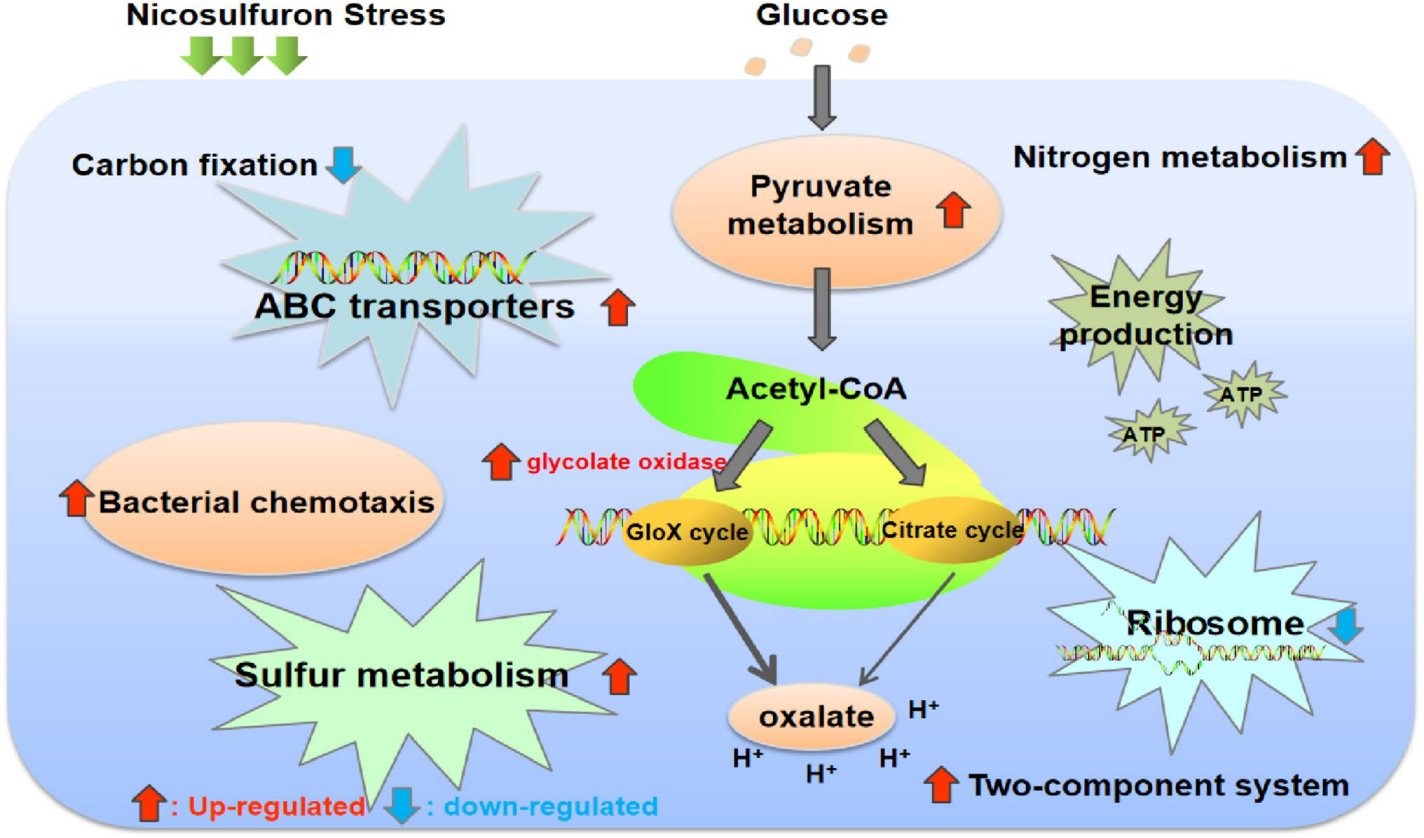
Transcriptomic responses of *Pseudomonas nicosulfuronedens* LAM1902 to nicosulfuron. : the number of up-regulated genes > the number of down-regulated genes, : the number of up-regulated genes < the number of down-regulated genes.

In addition, nine DEGs related to “bacterial chemotaxis” were revealed with KEGG annotation, including eight up-regulated genes and one down-regulated gene in response to nicosulfuron (Table 1). The genes of “bacterial chemotaxis” were the main part of the bacterial adjustment system. Microbes express“bacterial chemotaxis” genes to induce their migration towards the pollutants as part of a resistance mechanism to exposure to contaminated environments. Microbes would therefore become stronger by promoting these adaptive and degrading abilities^43^. Zhao showed that genes related to “bacterial chemotaxis” were up-regulated under levofloxacin stress using qPCR and found that play a pivotal role in levofloxacin resistance of strain *Stenotrophomonas maltophilia*^59^. Similarly, it is possible that the up-regulated genes of “bacterial chemotaxis” may provide strain LAM1902 with stronger adaptability to the exposure of nicosulfuron stress. Furthermore, 20 up-regulated genes associated with flagella assembly were determined in strain LAM1902, which suggests that this strain can target and degrade nicosulfuron and/or adapt to the contaminated environment. Data revealed several metabolism pathways associated with C- and N- metabolism, glycolysis, TCA cycle, and bacterial chemotaxis were affected by nicosulfuron, which would provide a better understanding of microbial metabolic networks of microorganisms in harsh environments (Fig. 5).

The involvement of genes and enzymes during the degradation of sulfonylurea herbicides has been discussed elsewhere^13,60,61^. However, the genes involved in the degradation of nicosulfuron were rarely studied^30^. The strain LAM1902 transcriptome results were used here to investigate the possible nicosulfuron degradation genes. It was interesting that the involvement of hydrolase and monooxygenase in sulfonylurea herbicide degradation^43^ was not observed in the transcription process of strain LAM1902 of our study. The genes associated with cytochromes P-450 were down-regulated (YG) compared to the control groups (NG), and only one gene encoding carboxylesterase was detected, whereas there was no significant difference between YG and NG groups (*p* > 0.05) (Table 2). On the other hand, the top 10 genes with significant differences between YG and NG groups (Table S5) were generally related to the metabolism of glucose and amino acids. For example, LAM1902_ctg012_orf00043 (glyceraldehyde-3-phosphate dehydrogenase) and LAM1902_ctg001_orf00053 (N-acetyltransferase) were related to glycolysis metabolism. LAM1902_ctg029_orf00075 (arginine–tRNA ligase), LAM1902_ctg016_orf00128 (urocanate hydratase), and LAM1902_ctg016_orf00131 (histidine ammonia-lyase) were related to amino acid metabolism. These data were consistent with the results from a previous metabolomics study^35^, showing that many significant metabolites were also responsible for glucose metabolism (D-glucose, trehalose) and amino acid metabolism (hydroxypyruvic acid, 3-aminoisobutanoic acid). Overall, results suggested that nicosulfuron stimulation has a strong effect on the glucose metabolism of strain LAM1902.

**Table 2:** Genes potentially involved in the nicosulfuron degradation by strain LAM1902. YG: conducted with LAM1902 in the presence of nicosulfuron; NG: conducted with LAM1902 without nicosulfuron.

Gene_ID	count_YG	count_NG	*p*-value	Annotation
LAM1902_ctg043_orf00049	82	20	0.19579705	carboxylesterase
LAM1902_ctg048_orf00055	140	505	3.59E-25	cytochrome P450
LAM1902_ctg003_orf00224	118	46	0.000204088	glycolate oxidase subunit GlcE
LAM1902_ctg003_orf00221	145	66	0.000681792	glycolate oxidase subunit GlcF

### Organic acid metabolism analysis of the response of strain LAM1902 to nicosulfuron stress

**Figure 6:**
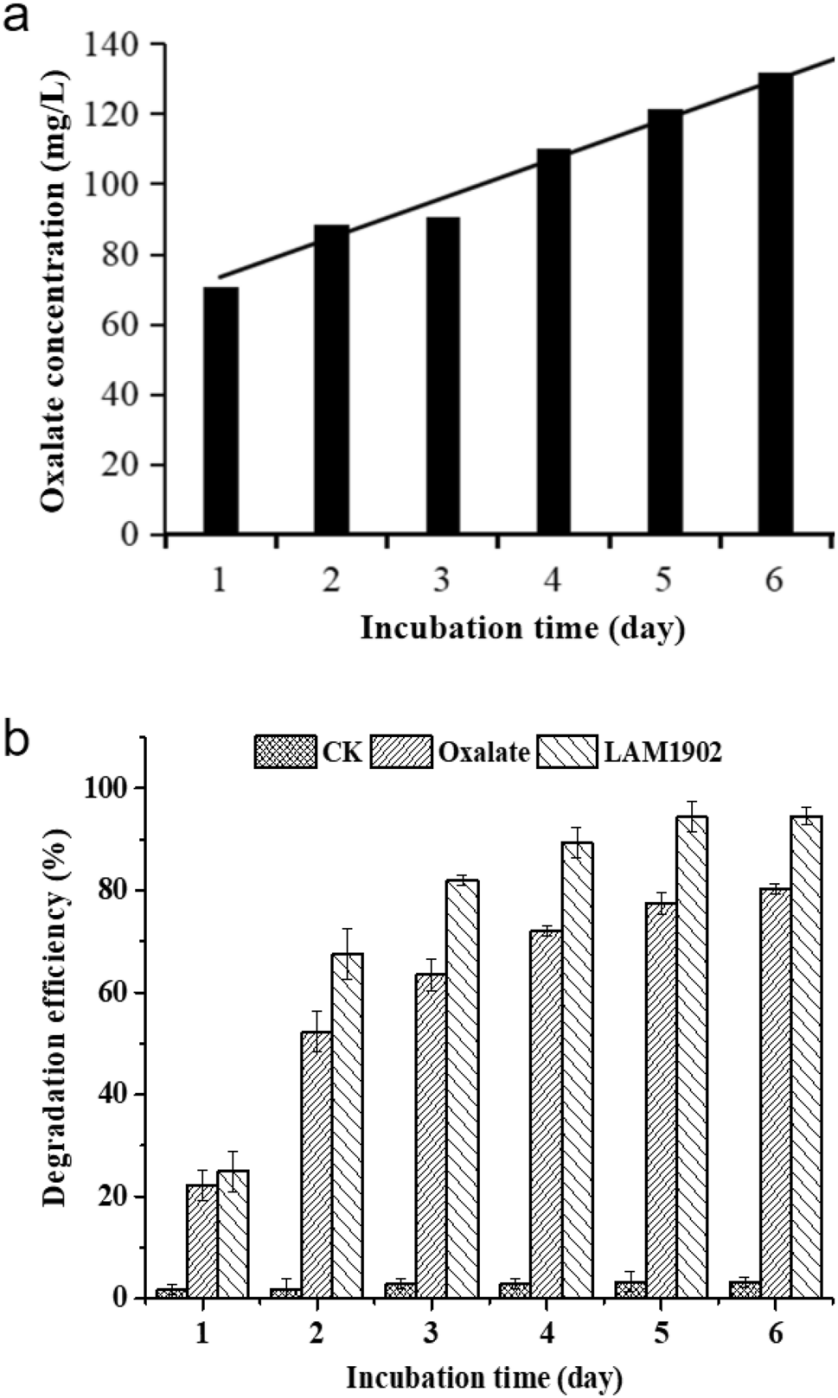
(**a**) The production of oxalic acid by strain LAM1902 during nicosulfuron biodegradation (after 6 days of incubation). (**b**) Comparison of nicosulfuron (50 mg/L) degradation efficiency under abiotic conditions (CK), in the presence of oxalic acid (200 mg/L) (Oxalate), and under biotic conditions (LAM1902).

Strain LAM1902 produces oxalic acid during nicosulfuron degradation reaching a concentration of 131 mg/L after 6 days (Fig. 6a). Notably, oxalic acid was also detected in the negative control (without nicosulfuron). To check its effect, we compared the nicosulfuron degradation by oxalic acid (200 mg/L) with that by the strain LAM1902 in GSM (Fig. 6b). The results showed oxalic acid could increase nicosulfuron degradation. Two synthetic pathways for oxalic acid in LAM1902 cells were proposed: the glyoxylate cycle and oxaloacetate synthesis pathways. Among them, glycolate oxidase and oxaloacetate lyase are the key enzymes in the synthesis pathway of oxalate^62^. The expression of genes encoding glycolate oxidase and oxaloacetate lyase under the nicosulfuron condition (YG group) with those in the control condition (NG group) were compared and analyzed. Under the nicosulfuron condition, glycolate oxidase was encoded by two up-regulated genes (LAM1902_ctg003_orf00224 and LAM1902_ctg003_orf00221, *p* ≤ 0.05), whereas DEGs encoding oxaloacetate lyase were not detected (Table 2). These results indicated that nicosulfuron exposure showed a positive effect on the synthesis of glycolate oxidase, which would then promote the production of oxalic acid. Then oxalic acid was excreted out of the cell to degrade nicosulfuron and relieve the toxicity of nicosulfuron. The present results indicate that the synthesis of oxalic acid by strain LAM1902 under nicosulfuron stress was mainly performed by the glyoxylate cycle pathway.

## Conclusion

In the present study, strain *P. nicosulfuronedens* LAM1902 can degrade several sulfonylurea herbicides (nicosulfuron, chlorimuron-ethyl, and cinosulfuron, 50 mg/L) in the incubation medium. In addition, the strain LAM1902 can tolerate and degrade nicosulfuron until 500 mg/L nicosulfuron exposure. This strain could be used as a mechanistic tool to extrapolate results from laboratory bioassays (liquid condition) to the field (soil condition) to better understand the bioremediation of sulfonylurea herbicide residues in the real environment. Moreover, this is the first report describing the use of transcriptomic analyses for identifying the molecular mechanism(s) of nicosulfuron-degrading bacterial strains. We provide here a theoretical basis for the development of future nicosulfuron biodegradation strategies for the treatment of nicosulfuron-contaminated soil. Further study into the ability of strain LAM1902 to degrade nicosulfuron in soil is warranted. And greater attention should be paid to the molecular mechanisms of nicosulfuron toxicity and environmental risk assessment of the transformation products during nicosulfuron degradation under actual conditions.

## Materials and methods

### Chemicals, reagents, and culture media

Analytical HPLC-grade nicosulfuron (CAS 111991-09-4; 1-(4,6-dimethoxypyrimidin-2-yl)-3-(3-dimethylcarbamoyl-2-pyridylsulfonyl)urea), chlorimuron-ethyl (CAS 90982-32-4; ethyl 2-(4-chloro-6-methoxy-2-pyrimidinylcarbamoylsulfamoyl)benzoate), and cinosulfuron (CAS 94593-91-6; 1-(4,6-dimethoxy-1,3,5-triazin-2-yl)-3-(2-(2-methoxyethoxy)phenylsulfonyl)urea) were purchased from the Aladdin Industrial Shanghai Co., Ltd. (China).

The Luria Bertani (LB) medium containing (in g/L) 10.0 peptone and 5.0 yeast extract (both obtained from Beijing Aoboxing Bio-tech Ltd), 10.0 NaCl, and pH 7.0 was used for culturing strain LAM1902. The sulfonylurea herbicide degradation studies using strain LAM1902 were carried out according to Li et al.^35^ using glucose supplemented medium (GSM) (pH 7.0) containing (in g/L) 1.0 NH_4_Cl, 1.0 NaH_2_PO_4_·12H_2_O, 0.5 KH_2_PO_4_, 0.2 MgSO_4_·12H_2_O, and 20 µL trace element solution, 5 g/L glucose, and 50 mg/L of nicosulfuron, chlorimuron-ethyl or cinosulfuron in distilled water^26^. The trace element solution (g/L; pH 7.0) contained: 5.5 CaCl_2_, 50.0 EDTA, 1.1 (NH_4_)6MoO_2_·4H_2_O, 5.0 FeSO_4_·7H_2_O, 2.2 ZnSO_4_, 5.1 MnCl_2_·4H_2_O, 1.6 CuSO_4_·5H_2_O, and 1.6 CoCl·6H_2_O. All the chemicals and reagents (at least analytical grade) were obtained from commercial sources, as listed in Supplementary Information (Table S1).

### Optimization of the nicosulfuron degradation conditions for *P. nicosulfuronedens* LAM1902

The degradation of nicosulfuron by strain LAM1902 was determined after 6 days of incubation, according to Li et al.^35^. Different incubation conditions included: different sources of carbon (1.0 g/L: sodium acetate, glycerol, glucose, sodium succinate, peptone, yeast extract, sucrose, and starch) and nitrogen (1.0 g/L: NH_4_Cl, (NH_4_)_2_SO_4_, NH_4_H_2_PO_4_, yeast extract, and peptone), increasing from pH 5 to pH 9, increasing temperatures from 15 °C to 45 °C, and using different incubation volumes (0, 1, 3, 5, 7, and 10%, v/v). Different initial nicosulfuron concentrations (10, 25, 50, 100, 200, or 500 mg/L GSM) were used at 30 °C on a rotary shaker at 150 rpm. Based on these nicosulfuron degradation optimization studies, the strain LAM1902 was incubated in 50 mg/L nicosulfuron in GSM at pH 6, at 30 °C, and with an incubation volume of 5%, unless otherwise specified. The residual nicosulfuron concentrations (determined by using HPLC) were determined daily. All treatments were performed in triplicate. Details can be found in the Supplementary Information section (Table S2).

### Sulfonylurea herbicide degradation studies using *P. nicosulfuronedens* LAM1902

The degradation of nicosulfuron by strain LAM1902 was compared with chlorimuron-ethyl and cinosulfuron (50 mg/L) after 6 days of incubation at pH 6 and 30 °C, using a 5% incubation volume. The negative control group was incubated with autoclaved LAM1902 cells (abiotic control). The residual concentrations (determined using HPLC analysis) and cell growth (OD_600_) were determined daily for six days.

Chemical analyses of sulfonylurea herbicide concentrations were determined according to others^30,35^ using a high-performance liquid chromatography (HPLC) system (Agilent 1200, Waldbronn city, Germany) with a 10 µL injection volume, a C18 column (50 mm × 2.1 mm, 3.5 µm). For nicosulfuron analysis, the mobile phase was a mixture of acetonitrile/water/acetic acid (30/68/2, v/v/v), and a flow rate of 1.0 mL/min. The photodiode array detector had a wavelength of 210 nm, and the column temperature was 30 °C. Peak identification was based on our earlier paper^35^; and the HPLC peak retention time (*R*_*t*_) of nicosulfuron was 5.6 min, using the authentic nicosulfuron sample.

For chlorimuron analysis, the mobile phase was a mixture of methanol/water/acetic acid (67/32/1, v/v/v, the flow rate of 1.0 mL/min) and a photodiode array detector set at 236 nm, and a column temperature of 30 °C. For the cinosulfuron studies, the HPLC used a photodiode array detector at 240 nm, with a column temperature of 28 °C; the mobile phase was a mixture of acetonitrile/methanol/water/acetic acid (45/15/40/0.1, v/v/v, flow rate of 1.0 mL/min). The respective *R*_*t*_ of chlorimuron-ethyl and cinosulfuron were respectively 10 min and 6 min, using their authentic reference samples. The % efficiencies of strain LAM1902 to degrade nicosulfuron, chlorimuron-ethyl, and cinosulfuron were calculated. The key metabolites of nicosulfuron (ASDM and ADMP) were also determined by HPLC; the *Rt* of these metabolites (*Rt* of ASDM = 1.78 min and *Rt* of ADMP = 2.59 min) were similar to that of nicosulfuron.

### Identification of oxalic acid production and the degradation effect on nicosulfuron

The oxalic acid produced by strain LAM1902 in the GSM medium was identified by HPLC (using the same column as that described above) with an injection volume of 10 µL. The mobile phase was a mixture of ammonium dihydrogen phosphate (0.02 mM, pH 2, adjusted with H_3_PO_4_) and methanol (85:15 by volume, flow rate of 1.0 mL/min). The photodiode array detector was set at 210 nm, and the column temperature was 30 °C. The HPLC peak retention time (*Rt*) of oxalic acid was 5.5 min. The effects of oxalic acid (200 mg/L) on nicosulfuron (50 mg/L) degradation were by comparing the results of three experiments: (1) the control group (CK) corresponding to the abiotic conditions (nicosulfuron in GSM), (2) the oxalate-supplemented GSM group (nicosulfuron + oxalic acid), and (3) the biodegradation group (nicosulfuron + LAM1902 in GSM). All treatments were performed in triplicate.

### Total RNA isolation and sequencing

*Pseudomonas nicosulfuronedens* LAM1902 was incubated in the GSM medium at 3% (v/v) (i.e., 3 mL strain LAM1902 plus 97 mL GSM) in presence of 50 mg/L nicosulfuron, which served as the experimental group (YG). In parallel, the culture was grown in the absence of nicosulfuron and was considered a control group (NG)^63^. After culturing for 3 d (the logarithmic growth phase of strain LAM1902) at 30 °C and 160 rpm in the dark, the samples were centrifuged at 8000 rpm (Sigma, Germany) for 10 min, 4 °C. The pellet was washed three times with 130 mM phosphate buffer (pH 7.2), then quickly frozen in liquid nitrogen, and stored at -80 °C. All experiments were conducted in triplicate.

Total RNA was extracted using commercial kits following the manufacturer’s instructions (Ambion, Foster City, CA). RNA degradation and contamination were monitored on 1% agarose gels. The RNA quantity was measured using Qubit 2.0 (Thermo Fisher Scientific, MA, USA) and Nanodrop One (Thermo Fisher Scientific, MA, USA). RNA integrity was detected using the Agilent 2100 system (Agilent Technologies, Waldbronn, Germany).

Whole mRNAseq libraries were generated by Guangdong Magigene Biotechnology (Guangzhou, China) using the NEB Next Ultra Directional RNA Library Prep Kit for Illumina (New England Biolabs, MA, USA) following the manufacturer’s recommendations. Briefly, the 16S ribosomal RNA (rRNA) transcripts in total RNA samples were reduced by the Ribo-Zero rRNA removal kit. Fragmentation was carried out using the NEBNext RNA First-Strand Synthesis reaction buffer. The first-strand cDNA was synthesized using a random hexamer primer and M-MuLV Reverse Transcriptase (RNase H). For synthesizing the second strand of cDNA, a chain-specific library was constructed by replacing dTTP with dUTP to improve the accuracy of the results. The remaining overhangs were converted into blunt ends via exonuclease/polymerase activities. After adenylation of 3’ ends of DNA fragments, NEBNext Adaptor with its hairpin loop structures was ligated in preparation for hybridization. To choose cDNA fragments of preferentially 150 ~ 200 bp in length, the fragments were selected using AMPure XP beads (Beckman Coulter, Beverly, USA). PCR was then performed with Phusion High-Fidelity DNA polymerase, Universal PCR primers, and Index (X) Primer (Premier Biosoft International, Palo Alto, USA). PCR products were purified with AMPure XP beads, and the library insert size was assessed on the Agilent 2100 system (Agilent Technologies, Waldbronn, Germany). The clustering of the index-coded samples was performed on a cBot Cluster Generation System. The sequencing library was operated and sequenced on the Illumina Hiseq Xten platform by Guangdong Magigene Biotechnology (Guangzhou, China). Libraries for transcriptome analysis were established by using RNA collected from the YG and NG groups.

### Analysis of RNA-Seq data

The *fastq* format raw data were treated with Trimmomatic (v.0.36) to obtain the clean reads^64^, with mapping to NCBI Rfam databases for removing the rRNA sequences by Bowtie2 (v2.33). The residual mRNA sequences were compared with the reference genome using Hisat2 (2.1.0)^65^. The read count and function of each gene were obtained using HTSeq-count (v0.9.1). The reads per kilobase per million for each gene were used to compare the expression level of genes among the experimental and control groups. The read count of each gene (acquired from HTSeq-count) was mainly used for analyzing the differential expression. The DEGs between two groups were identified using the edgeR (v3.16.5)^66–68^. The GO and KEGG enrichment analyses of DEGs were performed by the cluster Profiler software (v3.4.4) to analyze their potential biological pathways^69^.

### Statistical analyses

Data were analyzed using one-way analysis of variance (ANOVA) and SPSS software (ver.18.0, SPSS Inc., Chicago, IL, United States). The resulting *p*-value was adjusted for judging the false discovery rate. Genes with the false discovery rate correction, *p* ≤ 0.05, and |log_2_ (fold change)|≥ 1 were taken as the candidates of DEGs^43,70^. The GO terms and KEGG pathways with *p* ≤ 0.05 were identified as significantly enriched by DEGs.

## Supplementary Information

Supplementary Information.

## Data Availability

All the RNA-seq data have been deposited in the NCBI Sequence Read Archive (SRA) database (Accession Number: PRJNA785098).
