# Supplementary material for: Transcriptomic response of Pseudomonas nicosulfuronedens LAM1902 to the sulfonylurea herbicide nicosulfuron
Source: Sci Rep. 2022 Aug 11;12:13656. doi: 10.1038/s41598-022-17982-7 (PMC9372043; doi:10.1038/s41598-022-17982-7)
Supplement: Supplementary file 1 — Supplementary Information. [file 41598_2022_17982_MOESM1_ESM.pdf]

SUPPLEMENTARY INFORMATION

Transcriptomic response of *Pseudomonas nicosulfuronedens* LAM1902 to the sulfonylurea herbicide nicosulfuron

Miaomiao Li[a,b]†, Qingqing Li[b]†, Jun Yao[a], Geoffrey Sunahara[a,c], Robert Duran[e], Qinghua Zhang[d], Zhiyong Ruan[b,*]

[a] School of Water Resource and Environment, Research Center of Environmental Science and Engineering, China University of Geosciences (Beijing), 29 Xueyuan Road, Haidian District, 100083, Beijing, China

[b] Institute of Agricultural Resources and Regional Planning, Chinese Academy of Agricultural Sciences, Beijing 100081, China

[c] Department of Natural Resource Sciences, McGill University, 21111 Lakeshore Drive, Ste-Anne-de-Bellevue, Quebec, H9X 3V9, Canada

[d] College of Bioscience and Engineering, Jiangxi Agricultural University, Nanchang 330045, PR China

[e] Universite de Pau et des Pays de l'Adour, UPPA/E2S, IPREM CNRS 5254, Pau, France

* Corresponding authors:

Zhiyong Ruan (E-mail: ruanzhiyong@caas.cn)

Institute of Agricultural Resources and Regional Planning, Chinese Academy of Agricultural Sciences, 100083, Beijing, China

† These authors contributed equally to this work.

Supplementary Information

Fig. S1. Degradation of nicosulfuron by *Pseudomonas nicosulfuronedens* LAM1902 under different initial concentration of nicosulfuron (mg/L) at 6 days.

Fig. S2. Chemical structures of selected sulfonylurea herbicides. Nicosulfuron (left), chlorimuron-ethyl(middle) and cinosulfuron (right).

Fig. S3. A principal component analysis (PCA) was performed to visualize the differences in the two groups (NG, conducted with LAM1902 without nicosulfuron, and YG, conducted with LAM1902 in the presence of nicosulfuron). All groups were conducted in triplicate.

Fig. S4. Heat map of the top 30 expression genes in two groups. “NG” represents the control group, without nicosulfuron; “YG” represents the experimental group, with nicosulfuron. All groups were conducted in triplicate.

Fig. S5. GO level2 classification of differentially expressed genes during nicosulfuron degradation. The up-regulated DEGs were colored red, and down-regulated DEGs were colored green.

Table S1 Suppliers and purities of chemicals.

Table S2 Incubation conditions used for each optimization experimental series.

Table S3 Statistical analysis of transcriptome sequencing in two groups.

Table S4 The KEGG annotation and statistical analysis of DEGs involved in nicosulfuron degradation ($p \leq 0.05$ and $|\log_2(\text{fold change})| \geq 1$). *p*. adjustment represent the correction of *p*-value.

Table S5 Top 10 genes with significant differences between YG and NG groups ($p \leq 0.05$).

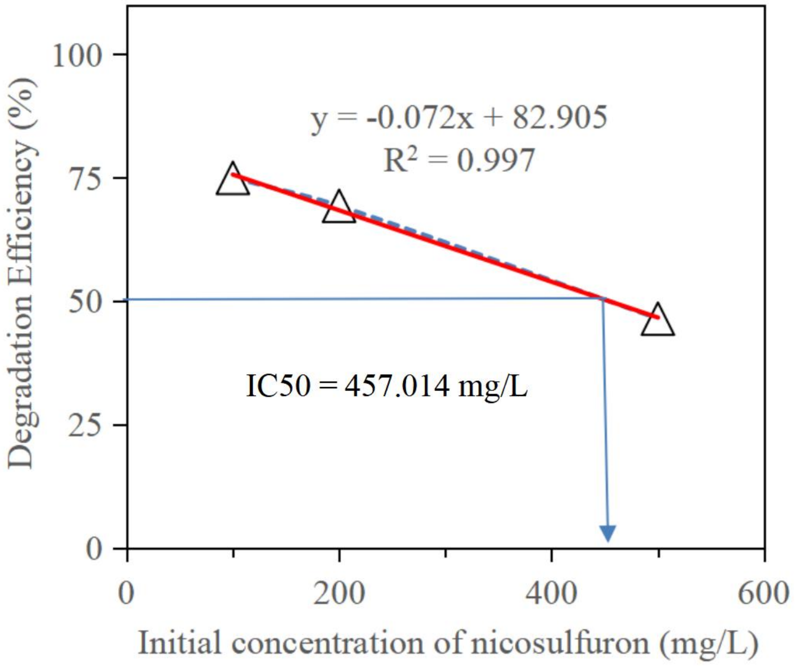

Fig. S1. Degradation of nicosulfuron by *Pseudomonas nicosulfuronedens* LAM1902 under different initial concentration of nicosulfuron (mg/L) at 6 days.

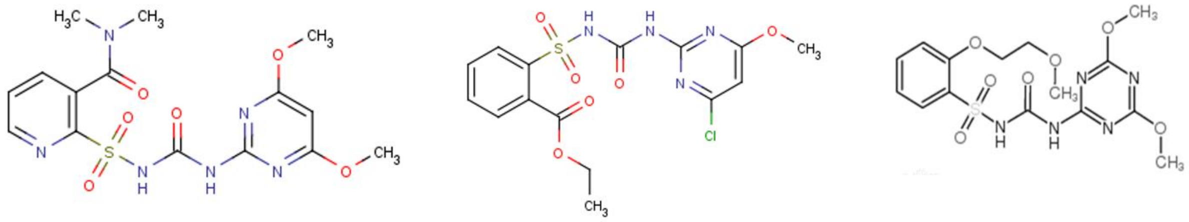

Nicosulfuron Chlorimuron-ethyl Cinosulfuron

Fig. S2. Chemical structures of selected sulfonylurea herbicides. Nicosulfuron (left), chlorimuron-ethyl(middle) and cinosulfuron (right).

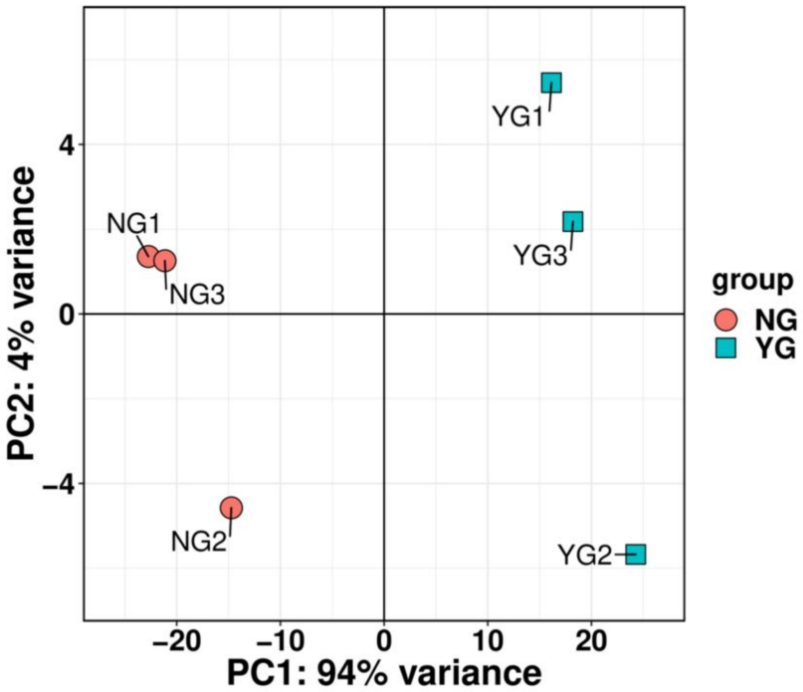

Fig. S3. A principal component analysis (PCA) was performed to visualize the differences in the two groups (NG, conducted with LAM1902 without nicosulfuron, and YG, conducted with LAM1902 in the presence of nicosulfuron). All groups were conducted in triplicate.

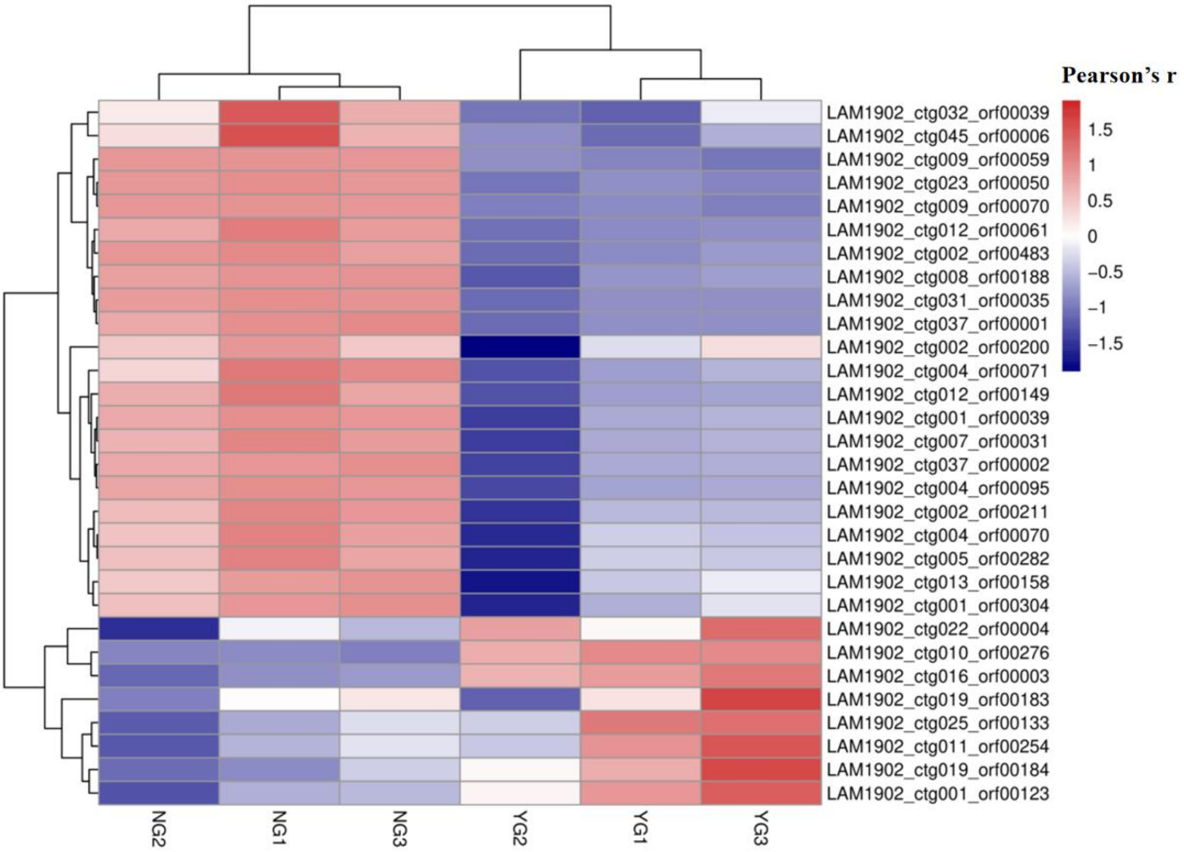

Fig. S4. Heat map of the top 30 expression genes in two groups. "NG" represent the control group, without nicosulfuron; "YG" represent the experimental group, with nicosulfuron. All groups were conducted in triplicate.

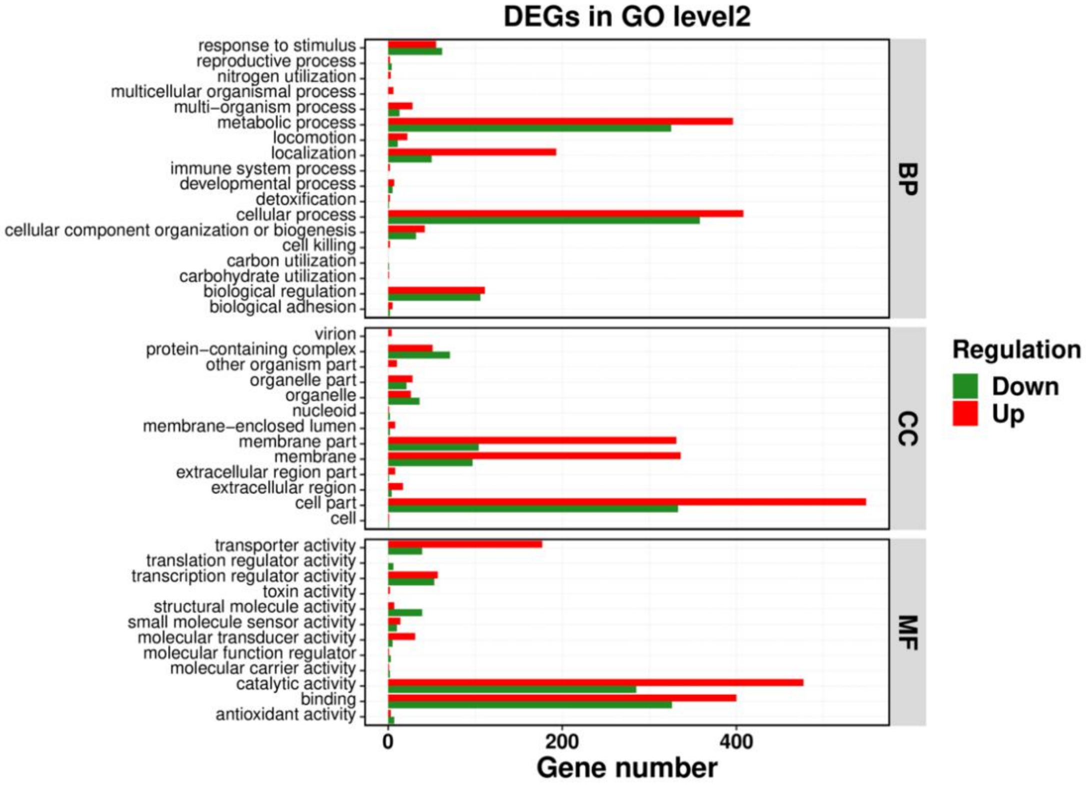

Fig. S5. GO level2 classification of differentially expressed genes during nicosulfuron degradation. The up-regulated DEGs were colored red, and down-regulated DEGs were colored green.

Table S1 The supplier and purity of chemicals.

Chemical	Supplier	Purity
peptone	Beijing Aoboxing Bio-tech Co.,LTD.	ND
yeast extract	Beijing Aoboxing Bio-tech Co.,LTD.	ND
glucose	Tianjin Guangfu Fine Chemical Research Institute	>96.0%
sodium acetate	Tianjin Fuchen Chemical Reagents Factory	>99.0%
glycerol	Xilong Scientific	>99.0%
sodium succinate	Tianjin Fuchen Chemical Reagents Factory	>99.0%
sucrose	Tianjin Fuchen Chemical Reagents Factory	>99.0%
starch	Tianjin Fuchen Chemical Reagents Factory	>90.0%
NaCl	Tianjin Fuchen Chemical Reagents Factory	>99.5%
NH_4Cl	Tianjin Fuchen Chemical Reagents Factory	>99.0%
$NaH_2PO4\cdot12H_2O$	Tianjin Fuchen Chemical Reagents Factory	>99.5%
KH_2PO4	Tianjin Fuchen Chemical Reagents Factory	>99.0%
$MgSO_4\cdot12H_2O$	Tianjin Fuchen Chemical Reagents Factory	>99.0%
EDTA	Xilong Scientific	>99.0%
$(NH_4)_6MoO_2\cdot4H_2O$	Tianjin Fuchen Chemical Reagents Factory	>99.0%
$FeSO_4\cdot7H_2O$	Tianjin Fuchen Chemical Reagents Factory	>99.0%
$ZnSO_4$	Tianjin Fuchen Chemical Reagents Factory	>99.5%
$MnCl_2\cdot4H_2O$	Tianjin Fuchen Chemical Reagents Factory	>99.0%
$CuSO_4\cdot5H_2O$	Sinopharm Chemical Reagent Co.,Ltd	>99.0%
$CoCl\cdot6H_2O$	Tianjin Fuchen Chemical Reagents Factory	>99.0%
$(NH_4)_2SO_4$	Tianjin Fuchen Chemical Reagents Factory	>99.0%
$NH_4H_2PO_4$	Tianjin Fuchen Chemical Reagents Factory	>99.0%

Table S2 Incubation conditions used for each optimization experimental series.

	Carbon sources	Nitrogen sources	pH	Incubation amount (%)	Temperature (°C)	Initial concentration of nicosulfuron (mg/L)
Carbon sources	—	NH_4Cl	7.0	5	30	50
Nitrogen sources	Glucose	—	7.0	5	30	50
pH	Glucose	NH_4Cl	—	5	30	50
Incubation amount	Glucose	NH_4Cl	7.0	—	30	50
Temperature	Glucose	NH_4Cl	7.0	5	—	50
Initial concentration of nicosulfuron	Glucose	NH_4Cl	7.0	5	30	—

Table S3 Statistical analyses of transcriptome sequencing in two groups.

**Sample**	**Clean paired reads**	**Q20 (%)**	**Q30 (%)**	**GC content (%)**	**Total mapped reads**
NG1	10160972	98.7	95.25	59.84	9424292
NG2	11091190	98.86	95.70	60.24	10352445
NG3	9579072	98.61	95.01	60.22	8832320
YG1	12073981	98.79	95.51	59.61	11214042
YG2	8513869	98.64	95.10	59.06	7413664
YG3	9913018	98.73	95.73	59.81	9052267

Table S4 The KEGG annotation and statistical analyses of DEGs involved in nicosulfuron degradation ($p \leq 0.05$ and $|\log_2(\text{fold change})| \geq 1$). *p*.adjust represent the correction of *p*-value.

**KEGG annotation**	***p*-value**	***p*.adjust**	**Gene count**
ABC transporters	7.32E-11	1.46E-08	115
Sulfur metabolism	1.05E-08	1.05E-06	49
Ribosome	1.83E-07	1.22E-05	36

Table S5 Top 10 genes with significant differences between YG and NG groups ($p \leq 0.05$).

Gene_ID	count_YG	count_NG	Annotation
LAM1902_ctg010_orf00276	45745	28	sulfate export transporter
LAM1902_ctg016_orf00128	3883	58	urocanate hydratase
LAM1902_ctg016_orf00134	6848	353	formimidoylglutamate deiminase
LAM1902_ctg016_orf00131	2983	408	histidine ammonia-lyase
LAM1902_ctg012_orf00043	14439	2192	glyceraldehyde-3-phosphate dehydrogenase
LAM1902_ctg038_orf00074	3333	929	D-amino acid dehydrogenase
LAM1902_ctg001_orf00053	10552	3042	N-acetyltransferase
LAM1902_ctg016_orf00003	21336	6258	universal stress protein
LAM1902_ctg029_orf00075	12028	3883	arginine--tRNA ligase
LAM1902_ctg004_orf00183	1970	548	Ig-like domain repeat protein
